# Adverse Drug Events Associated with Low-Dose (10 mg) Versus High-Dose (25 mg) Empagliflozin in Patients Treated for Type 2 Diabetes Mellitus: A Systematic Review and Meta-Analysis of Randomized Controlled Trials

**DOI:** 10.1007/s13300-018-0399-z

**Published:** 2018-03-09

**Authors:** Xia Dai, Zu-chun Luo, Lu Zhai, Wen-piao Zhao, Feng Huang

**Affiliations:** 1 Department of Endocrinology, The First Affiliated Hospital of Guangxi Medical University, Nanning, 530021 Guangxi People’s Republic of China; 2 Department of Internal Medicine Education, The First Affiliated Hospital of Guangxi Medical University, Nanning, 530021 Guangxi People’s Republic of China; 3 Institute of Cardiovascular Diseases and Guangxi Key Laboratory Base of Precision Medicine in Cardio-cerebrovascular Diseases Control and Prevention, The First Affiliated Hospital of Guangxi Medical University, Nanning, 530021 Guangxi People’s Republic of China

**Keywords:** Adverse drug events, Empagliflozin, Hypoglycemia, Oral hypoglycemic agents, Type 2 diabetes mellitus, Urinary tract infections

## Abstract

**Introduction:**

Empagliflozin is a new, emerging oral hypoglycemic agent (OHA) which has shown significant benefits in type 2 diabetes mellitus (T2DM) patients with cardiovascular disease. In this analysis, our aim was to systematically compare the adverse drug events (ADEs) associated with a low (10 mg) versus a high (25 mg) dose of empagliflozin as (1) monotherapy, (2) as an add-on to other OHAs, and (3) as an add-on specifically to metformin, in patients who were treated for T2DM.

**Methods:**

This was a systematic review and meta-analysis of randomized controlled trials that compared empagliflozin 10 mg versus 25 mg in patients who were treated for T2DM and which reported adverse drug reactions as their clinical endpoints. Statistical analysis was carried out using the latest version of the RevMan software (ver. 5.3) whereby odds ratios (OR) and 95% confidence intervals (CI) were generated.

**Results:**

Eight trials with a total number of 8514 patients treated for T2DM were included in this meta-analysis and systematic review, of whom 4261 patients received 10 mg empagliflozin and 4253 patients received 25 mg empagliflozin. Our results showed that there were no significant differences between the patients with T2DM receiving 10 empagliflozin and those receiving 25 mg empagliflozin in terms of drug-related adverse effects (OR 1.06, 95% CI 0.93–1.21; *P* = 0.40, *I*^2^ = 0%), adverse events leading to drug discontinuation (OR 0.99, 95% CI 0.86–1.14; *P* = 0.87, *I*^2^ = 0%), and serious adverse events (OR 1.06, 95% CI 0.95–1.18; *P* = 0.31, *I*^2^ = 0%) when empagliflozin was provided as monotherapy or as an add-on to other anti-diabetic medications. The same results were obtained when empagliflozin was used as an add-on to metformin or as monotherapy. The duration of the follow-up periods did not affect the results. However, the incidence of genital and urinary tract infections (UTIs) was significantly higher in female patients than in male patients with 10 or 25 mg empagliflozin.

**Conclusions:**

The incidence of ADEs was not significantly different in T2DM patients receiving 10 versus 25 mg empagliflozin as monotherapy or as add-on to metformin or other anti-diabetic drugs during a shorter or longer follow-up period. However, genital and UTIs were more common in female patients with T2DM irrespective of empagliflozin dosage.

## Introduction

Type 2 diabetes mellitus (T2DM) is remains a disease which affects a major population of patients around the globe, but 2017–2018 is the beginning of a new era in which several newer oral hypoglycemic agents (OHAs) are expected to replace previously/currently used ones. However, further research is still required to elaborate and assess the advantages and disadvantages as well as the correct dosage of these new drugs. A systematic review and meta-analysis of patients with T2DM which compared treatment with 100 versus 30 mg canagliflozin, an oral medication for T2DM that helps the body excrete glucose via the urine, showed that there was no significant difference in terms of adverse drug events (ADEs) between the two different dosages of drug [1]. However, this clinically new notion should be examined with other newer OHAs.

Empagliflozin is a new, emerging OHA which has shown to have significant benefits in T2DM patients with cardiovascular disease [2]. The EMPA-HEART trial is an on-going study which is currently investigating the impact of empagliflozin on subclinical left ventricular dysfunctions and on the mechanisms which are involved in myocardial disease progression in patients with T2DM [3]. The EMBLEM trial is another multicenter, placebo-controlled, double-blind randomized trial which is still evaluating the effect of empagliflozin on endothelial function [4]. The results of these new trials are still under investigation. However, the adverse drug reactions of patients treated for T2DM with different dosages of empagliflozin have seldom been systematically compared in studies.

The aim of the analysis reported here was to systematically compare the ADEs that occurred in patients treated for T2DM with a low (10 mg) versus high (25 mg) dose of empagliflozin as (1) monotherapy, (2) as an add-on to other OHAs, and (3) as an add-on specifically to metformin.

## Methods

### Search Strategy

The first step in our meta-analysis and systematic review was to search the MEDLINE (including PubMed), EMBASE (http://www.sciencedirect.com), Cochrane library, the ClinicalTrials.gov registry (http://www.clinicaltrials.gov), and Google Scholar databases for relevant articles using the following search terms ‘empagliflozin’, ‘empagliflozin and diabetes mellitus’, ‘empagliflozin 10 verus 25 mg’, and ‘empagliflozin and type 2 diabetes mellitus’. Our search strategy was limited to English language publications and involved only human participants. The reference lists of articles assessed to be suitable for inclusion were also searched for relevant articles. 

### Inclusion and Exclusion Criteria

Studies were eligible for inclusion in the meta-analysis and systematic review if they met the following criteria: (1) exclusively a randomized controlled trial; (2) treatment with empagliflozin 10 versus 25 mg was compared in patients with T2DM; (3) events of interest were reported in the publication; (4) the follow-up period was ≥ 12 weeks.

Studies were excluded if: (1) they were not a randomized controlled trial; (2) they did not compare treatment with empagliflozin 10 versus 25 mg in patients with T2DM ; (3) events of interest were not reported in the trial; (4) they were duplicated studies; (5) they were pooled analyses of trials; (6) the follow-up period was < 12 weeks.

### Endpoints and Follow-Up Periods

Seven endpoints were assessed (for details see Table 1):

- drug-related adverse effects
- adverse events leading to drug discontinuation
- serious adverse events (any severe adverse event requiring assistance)
- death (due to ADEs)
- hypoglycemia (blood glucose level of ≤ 70 mg/dL or ≤ 3.9 mmol/L)
- urinary tract infections (UTIs): which were defined as infections of the urinary passage (upper and lower urinary tracts)
- genital infections: which were defined as infections of the genitalia especially on the external part

**Table 1:** Studies included in the meta-analysis and systematic review with outcomes and follow-up periods

Trials (first author)	Outcomes	Follow-up periods
Araki et al. [7]	Drug-related adverse effects, adverse events leading to drug discontinuation, serious adverse events, death, hypoglycemia, UTIs, genital infections	52 weeks
Ferrannini et al. [8]	Drug-related adverse effects, serious adverse events, adverse events leading to drug discontinuation, hypoglycemia, UTIs, genital infections	78 weeks
Haring et al. [9]	Drug-related adverse effects, adverse events leading to drug discontinuation, serious adverse events, death, hypoglycemia, UTIs, genital infections	24 weeks
Kadowaki et al. [10]	Drug-related adverse effects, adverse events leading to drug discontinuation, serious adverse events, death, hypoglycemia, UTIs, genital infections	52 weeks
Roden et al. [11]	Drug-related adverse effects, adverse events leading to drug discontinuation, serious adverse events, death, hypoglycemia, UTIs, genital infections	24 weeks
Softeland et al. [12]	Drug-related adverse effects, adverse events leading to drug discontinuation, serious adverse events, death, hypoglycemia, UTIs, genital infections	24 weeks
Takkanen et al. [13]	Drug-related adverse effects, adverse events leading to drug discontinuation, serious adverse events, death, hypoglycemia, UTIs, genital infections	12 weeks
Zinman et al. [14]	Drug-related adverse effects, adverse events leading to drug discontinuation, serious adverse events, death, hypoglycemia, UTIs, genital infections	3.1 years

*UTIs* Urinary tract infections

A short-term follow-up period of < 52 weeks and a longer follow-up period of ≥ 52 weeks were considered relevant to this analysis.

### Data Extraction and Quality Assessment

Data were extracted by five reviewers (XD, ZL, LZ, WZ and FH). The following data were extracted:

- type of study (priority was given to randomized controlled trials)
- names of first author
- year of publication
- time of patients’ enrollment
- total number of patients who were treated with 10 and 25 mg empagliflozin, respectively
- other anti-diabetic medications which were used
- duration of T2DM
- length of follow-up periods
- reported ADEs
- total number of male and female patients with UTIs
- range of glycated hemoglobin (HbA1c) in patients
- baseline features of participants in the trials
- methodological qualities of the trials.

The PRISMA reporting guideline was followed [5].

Quality assessment was carried out with reference to the features reported by the Cochrane Collaboration [6]. Grades (A–E) were given to each trial based on their methodological quality, with grade A suggesting very low risk of bias and grade E suggesting a high risk of bias.

### Statistical Analysis

Statistical analysis was carried out by the latest version of the RevMan version 5.3 software (Cochrane Collaboration, London, UK) whereby odds ratios (OR) and 95% confidence intervals (CI) were generated.

Heterogeneity was assessed by two statistical methods: (1) the *Q* statistical test, whereby a *P* value of ≤ 0.05 was considered to be statistically significant; (2) the *I*^2^ statistical test whereby the larger the value of *I*^2^, the higher the heterogeneity.

Two statistical models were used: (1) a fixed effects model (if *I*^2^ was < 50%); (2) a random effects model (if *I*^2^ was > 50%).

A sensitivity analysis was also carried out, whereby each trial was excluded from the main analysis, one by one, and a new analysis was carried out each time to observe any significant difference compared to the main results.

This analysis included a small volume of trials (< 10). Therefore, publication bias was assessed only by visually observing the funnel plots which were generated from the Revman software. Other methods to assess publication bias would be inappropriate with this small volume of studies.

### Compliance with Ethics Guidelines

This meta-analysis is based on previously conducted studies and does not contain any studies with human participants or animals performed by any of the authors. Hence, ethical approval was not required.

## Results

### Searched Outcomes

Our initial search of the five databases using the chosen search items identified 1146 articles. A first selection of these 1146 articles based on the titles and abstracts resulted in the elimination of 985 articles. The full text of the remaining 181 articles was assessed for eligibility, and 173 of these were eliminated for not satisfying the inclusion criteria as follows: non-randomized controlled trials (*n* = 112); did not compare 10 versus 25 mg empagliflozin (*n* = 5); did not report adverse events (*n* = 2); pooled analyses (*n* = 3); duplicated studies (*n* = 51) 

**Fig. 1:**
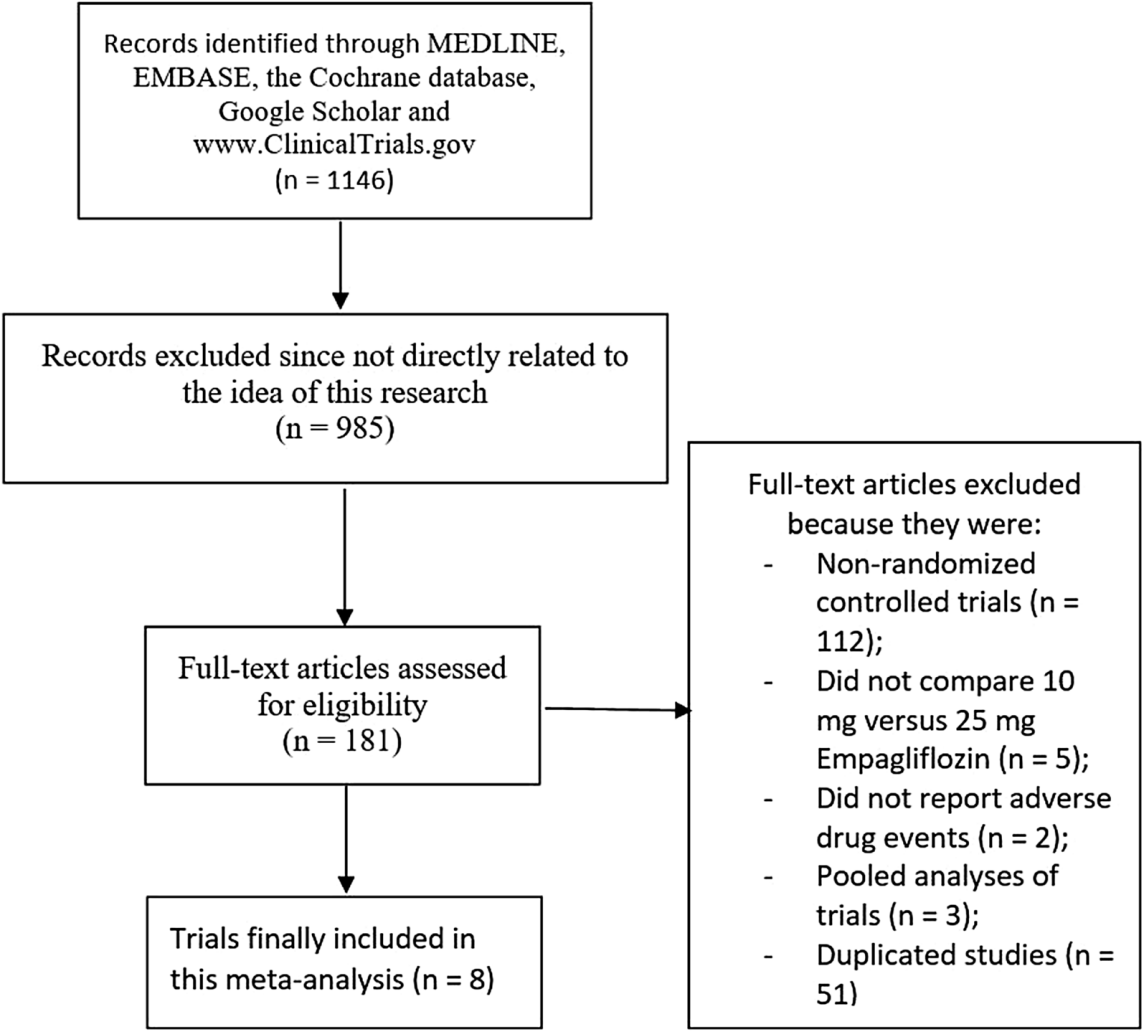
Flow diagram of the study selection process

Ultimately, only eight trials [7–14] satisfied all inclusion criteria and were included in the meta-analysis and systematic review. The flow chart for study selection is shown in Fig. 1.

### General Features of the Trials

Eight trials with a total number of 8514 patients treated for T2DM were included in this analysis (4261 patients received 10 mg empagliflozin and 4253 patients received 25 mg empagliflozin). The time period during which patients were enrolled in each study ranged from 2009 to 2015. The general features of the trials are listed in Table 2.

**Table 2:** General features of the trials

Trials	No of patients treated with 10 mg empagliflozin (*n*)	No of patients treated with 25 mg empagliflozin (*n*)	Enrollment period	Type of study	Bias risk grade^a^
Araki et al. [7]	548	549	2011–2013	RCT	B
Ferrannini et al. [8]	272	275	2009–2011	RCT	B
Haring et al. [9]	217	213	2010–2012	RCT	B
Kadowaki et al. [10]	267	265	2010–2014	RCT	B
Roden et al. [11]	224	223	2010–2012	RCT	A
Softeland et al. [12]	112	110	2013–2015	RCT	B
Takkanen et al. [13]	276	276	2011–2012	RCT	B
Zinman et al. [14]	2345	2342	2010–2013	RCT	A
Total no of patients (*n*)	4261	4253			

*RCT* Randomized controlled trials

^a^Grade A suggests very low risk of bias; grade E suggests a high risk of bias.

After referring to the bias risk assessment features suggested by the Cochrane collaboration, two trials were allotted a grade A, whereas the remaining trials were allotted a grade B, as shown in Table 2.

### Baseline Features of the Participants

The baseline characteristics of the participants of the eight studies are given in Table 3. At baseline the mean age of the patients ranged from 53.8 to 61.1 years, the percentage of male patients ranged from 48.1% to 75.7%, the body mass index ranged from 24.6 to 33.0 kg/m^2^, and the HbA1c ranged from 7.86% to 8.06% (Table 3). There was no significant difference in baseline features between those patients who received 10 mg empagliflozin and those who received 25 mg.

**Table 3:** Baseline features of the participants

Trials	Age (years)	Males (%)	HbA1c (%)	BMI (kg/m^2^)
	10 mg/25 mg	10 mg/25 mg	10 mg/25 mg	10 mg/25 mg
Araki et al. [7]	60.8/59.6	58.5/72.3	7.99/8.06	24.6/25.2
Ferrannini et al. [8]	59.5/59.5	48.1/52.7	7.89/8.00	28.9/28.1
Haring et al. [9]	55.5/55.6	58.0/56.0	7.94/7.86	29.1/29.7
Kadowak et al. [10]	57.3/57.9	75.7/74.0	7.94/7.93	25.4/25.4
Roden et al. [11]	56.2/53.8	63.0/65.0	7.87/7.86	28.3/28.2
Softeland et al. [12]	54.3/55.4	60.6/64.5	7.97/7.97	31.2/29.9
Takkanen et al. [13]	60.6/59.9	62.0/56.5	7.87/7.92	32.4/33.0
Zinman et al. [14]	61.1/61.1	73.1/73.9	8.06/8.05	26.8/26.5

*HbA1c* glycosylated hemoglobin, *BMI* Body Mass Index

The other anti-diabetic medications (sulphonylurea, metformin, thiazolidinedione, alpha-glucosidase inhibitor, glinide, or dipeptidyl-peptidase-4) and the duration of T2DM are given in Table 4.

**Table 4:** Other anti-diabetic drugs that were used in the trials included in the meta-analysis and systematic review

Trials	Other anti-diabetic agents which were used	Duration of T2DM (years)
Araki et al. [7]	Empagliflozin + sulphonyl urea or metformin or thiazolidinedione or alpha-glucosidase inhibitor or glinide or dipeptidyl-peptidase-4	1 to ≥ 10
Ferrannini et al. [8]	Empagliflozin mono-therapy and empagliflozin + metformin	1 to ≥ 5
Haring et al. [9]	Empagliflozin + metformin	1 to ≥ 10
Kadowaki et al. [10]	Empagliflozin monotherapy	–
Roden et al. [11]	Empagliflozin monotherapy	1 to ≥ 10
Softeland et al. [12]	Empagliflozin + linagliptin 5 mg and metformin	1 to ≥ 10
Takkanen et al. [13]	Empagliflozin monotherapy	1 to ≥ 10
Zinman et al. [14]	Empagliflozin + metformin and other anti-diabetic drugs (unspecified)	–

*T2DM* type 2 diabetes mellitus

### Comparison of the ADEs Associated with 10 versus 25 mg Empagliflozin as Monotherapy or Add-on to Other Anti-diabetic Medications in Patients with T2DM

**Fig. 2:**
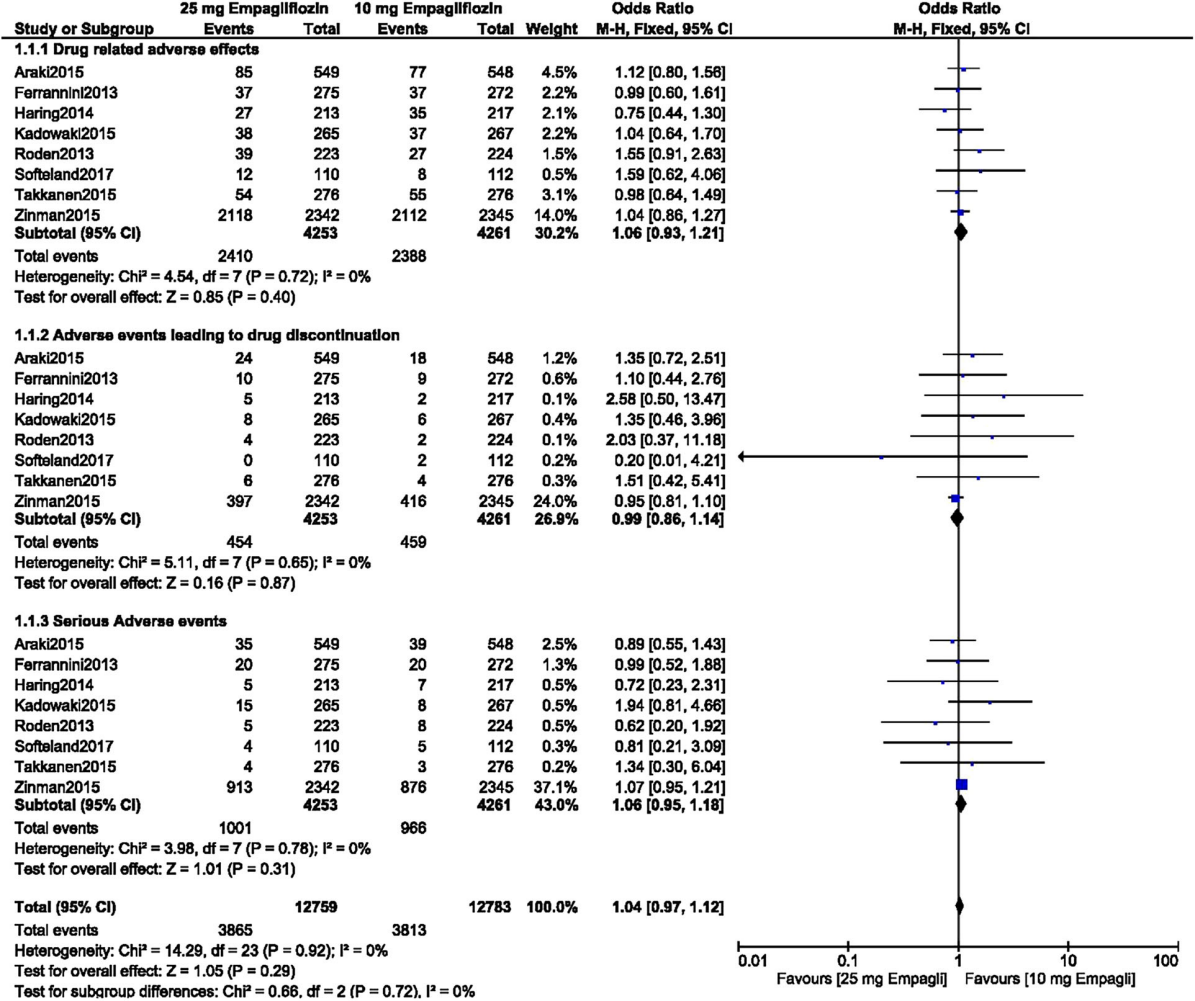

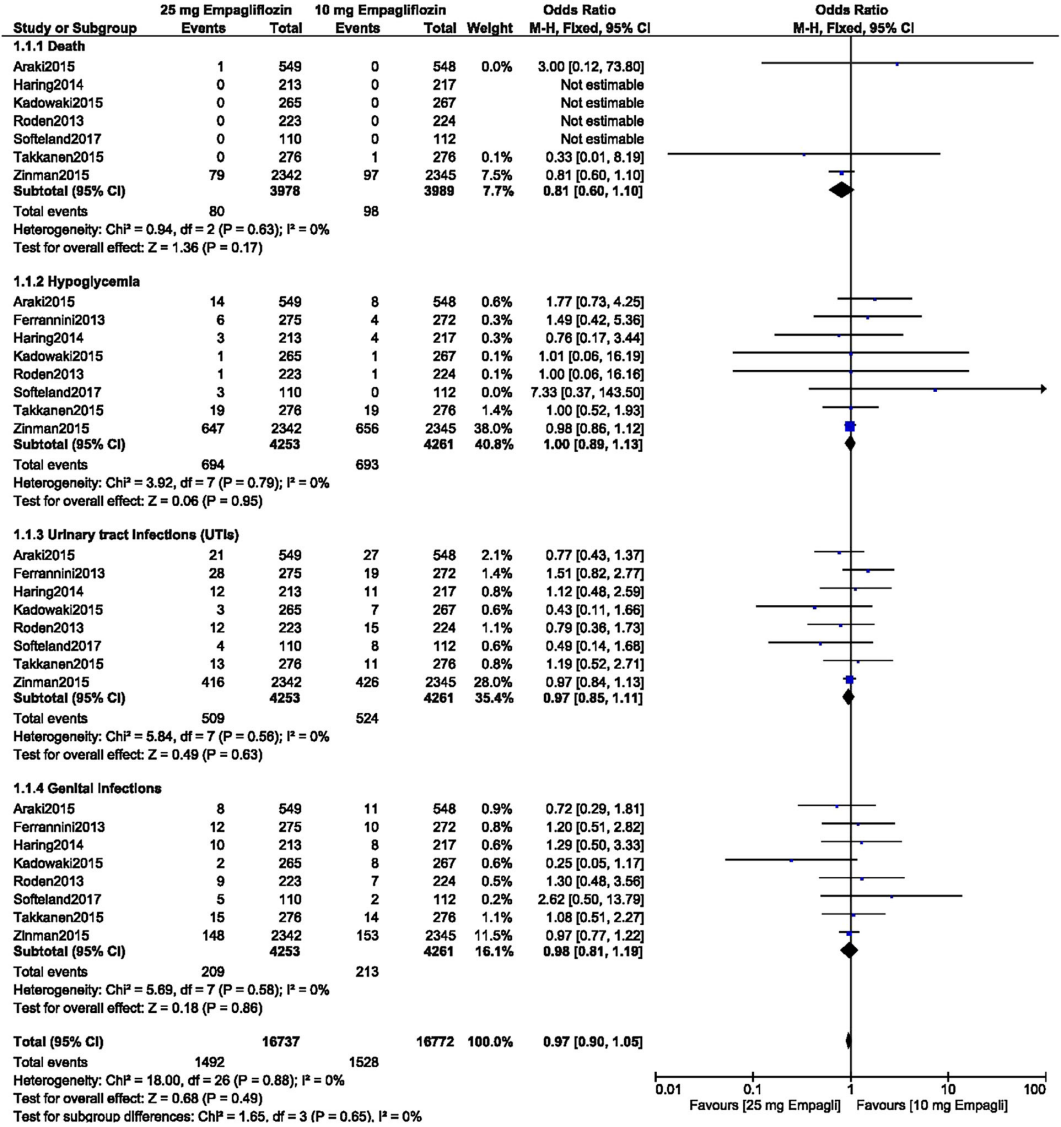
**a**,** b** Adverse drug events (**a**) and death, hypoglycemic episodes, urinary tract infections (*UTIs*), and genital infections (**b**) associated with treatment of patients with type 2 diabetes mellitus (T2DM) receiving 10 versus 25 mg empagliflozin as monotherapy or as add-on to other anti-diabetic medication.* CI* confidence interval,* M-H* Mantel-Haenszel method for calculating pooled odds ratio

Our results of our analysis show that there were no significant differences between the patients with T2DM receiving 10 empagliflozin and those receiving 25 mg empagliflozin in terms of drug-related adverse effects (OR 1.06, 95% CI 0.93–1.21; *P* = 0.40, *I*^2^ = 0%), adverse events leading to drug discontinuation (OR 0.99, 95% CI 0.86–1.14; *P* = 0.87, *I*^2^ = 0%), and serious adverse events (OR 1.06, 95% CI 0.95–1.18; *P* = 0.31, *I*^2^ = 0%) when empagliflozin was provided as monotherapy or as an add-on to other anti-diabetic medications, as shown in Fig. 2a.

 These patients with T2DM receiving 10 empagliflozin and those receiving 25 mg empagliflozin also showed similar manifestations of death (OR 0.81, 95% CI 0.60–1.10; *P* = 0.17, *I*^2^ = 0%), hypoglycemic episodes (OR 1.00, 95% CI 0.89–1.13; *P* = 0.95, *I*^2^ = 0%), UTIs (OR 0.97, 95% CI 0.85–1.11; *P* = 0.63, *I*^2^ = 0%), and genital infections (OR 0.98, 95% CI 0.81–1.19; *P* = 0.86, *I*^2^ = 0%), as shown in Fig. 2b.

### Comparison of the ADEs Associated with 10 Versus 25 mg Empagliflozin as Add-on to Metformin in Patients with T2DM

**Fig. 3:**
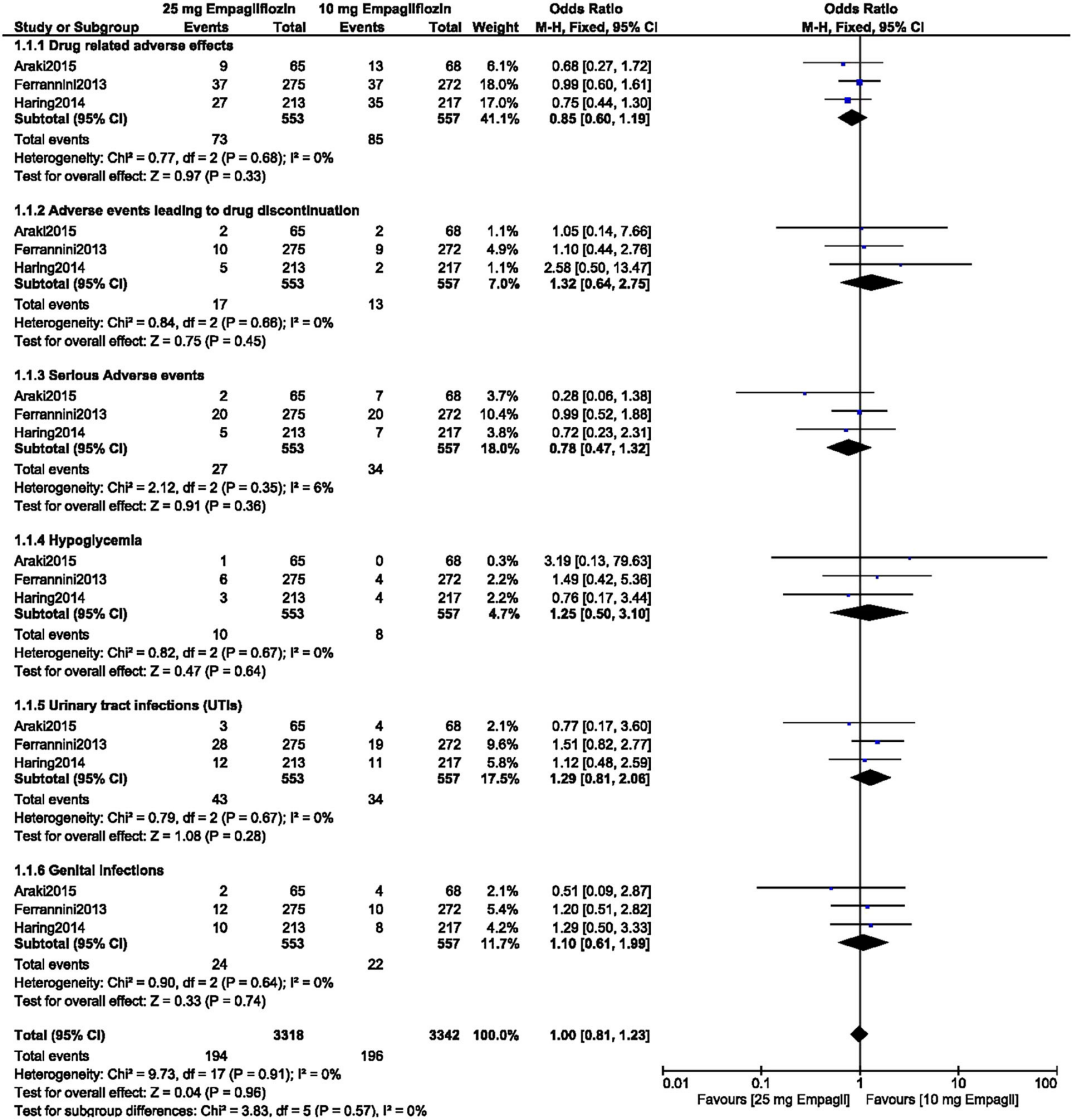
Adverse drug events associated with the treatment of patients with T2DM with 10 versus 25 mg empagliflozin as add-on to metformin

An analysis was carried out to compare the ADEs between these patients with T2DM which were associated with treatment with 10 versus treatment 25 mg empagliflozin as an add-on to metformin. Similar to the results reported above, there were no significant differences between the patients receiving 10 mg empagliflozin versus those receiving and 25 mg empagliflozin in terms of drug-related adverse effects (OR 0.85, 95% CI 0.60–1.19; *P* = 0.33, *I*^2^ = 0%), adverse events leading to drug discontinuation (OR 1.32, 95% CI 0.64–2.75; *P* = 0.45, *I*^2^ = 0%), serious adverse events (OR 0.78, 95% CI 0.47–1.32; *P* = 0.36, *I*^2^ = 6%), hypoglycemic episodes (OR 1.25, 95% CI 0.50–3.10; *P* = 0.64, *I*^2^ = 0%), UTIs (OR 1.29, 95% CI 0.81–2.06; *P* = 0.28, *I*^2^ = 0%), and genital infections (OR 1.10, 95% CI 0.61–1.99; *P* = 0.74, *I*^2^ = 0%), as shown in Fig. 3.

### Comparison of the ADEs Associated with 10 Versus 25 mg Empagliflozin Monotherapy in Patients with T2DM

**Fig. 4:**
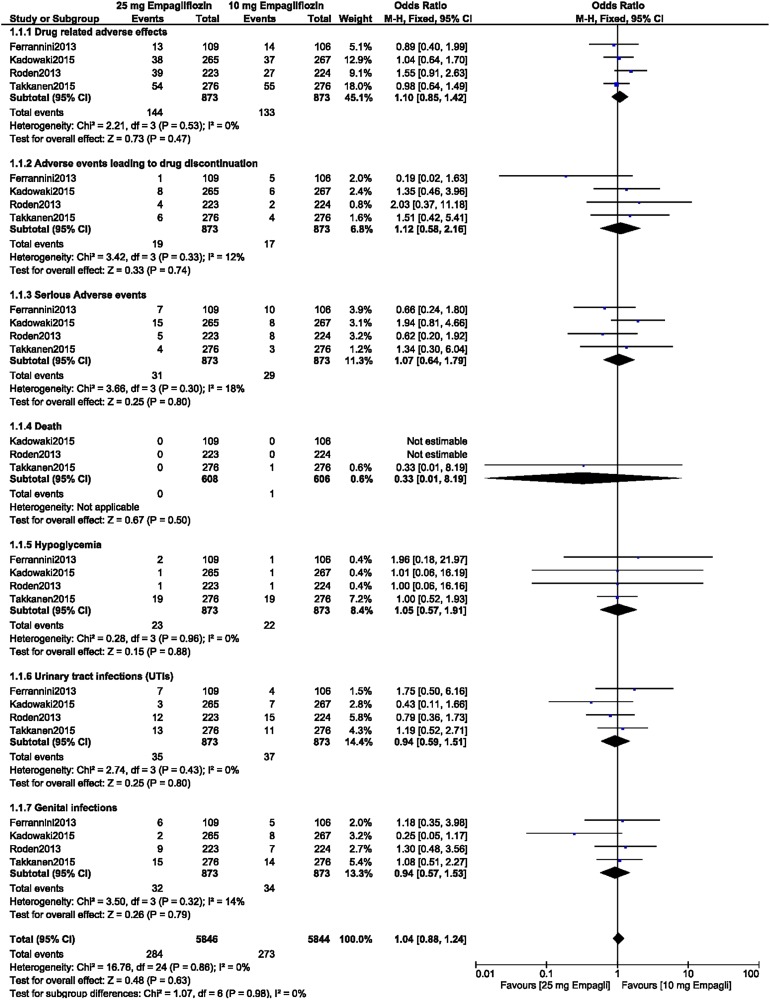
Adverse drug events associated with the treatment of patients with T2DM with 10 versus 25 mg empagliflozin monotherapy

Between patients with T2DM receiving 10 mg empagliflozin as monotherapy and those receiving 25 mg empagliflozin as monotherapy, there were no significant differences in terms of drug-related adverse effects (OR 1.10, 95% CI 0.85–1.42; *P* = 0.47, *I*^2^ = 0%), adverse events related to drug discontinuation (OR 1.12, 95% CI 0.58–2.16; *P* = 0.74, *I*^2^ = 12%), serious adverse events (OR 1.07, 95% CI 0.64–1.79; *P* = 0.80, *I*^2^ = 18%), death (OR 0.33, 95% CI 0.01–8.19; *P* = 0.50), hypoglycemic episodes (OR 1.05, 95% CI 0.57–1.91; *P* = 0.88, *I*^2^ = 0%), UTIs (OR 0.94, 95% CI 0.59–1.51; *P* = 0.80, *I*^2^ = 0%), and genital infections (OR 0.94, 95% CI 0.57–1.53; *P* = 0.79, *I*^2^ = 14%), as shown in Fig. 4.

### Comparison of the ADEs Associated with 10 Versus 25 mg Empagliflozin During a Short-Term Follow-Up Period of < 52 Weeks in Patients with T2DM

**Fig. 5:**
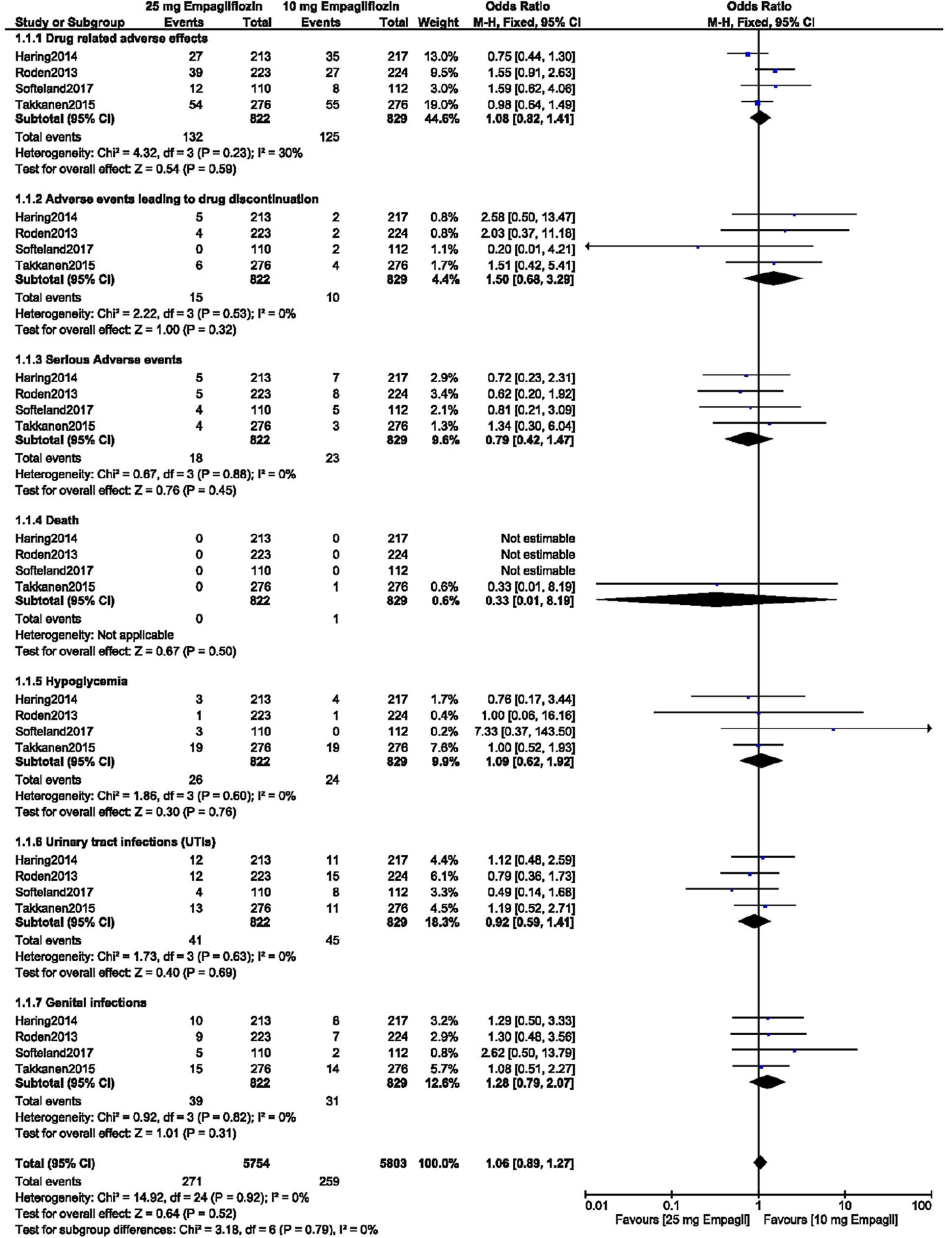
Adverse drug events associated with the treatment of patients with T2DM with 10 versus 25 mg empagliflozin during a short-term follow-up period of < 52 weeks

We also analyzed the data on treatment with 10 versus 25 mg empagliflozin according to length of the follow-up. During a short-term follow-up period of < 52 weeks, there were no significant differences in drug-related adverse effects (OR 1.08, 95% CI 0.82–1.41; *P* = 0.59, *I*^2^ = 30%), adverse events leading to drug discontinuation (OR 1.50, 95% CI 0.68–3.29; *P* = 0.32, *I*^2^ = 0%), serious adverse events (OR 0.79, 95% CI 0.42–1.47; *P* = 0.45, *I*^2^ = 0%), death (OR 0.33, 95% CI 0.01–8.19; *P* = 0.50), hypoglycemic episodes (OR1.09, 95% CI 0.62–1.92; *P* = 0.76, *I*^2^ = 0%), UTIs (OR 0.92, 95% CI 0.59–1.41; *P* = 0.69, *I*^2^ = 0%), and genital infections (OR 1.28, 95% CI 0.79–2.07; *P* = 0.31, *I*^2^ = 0%) between the T2DM patients receiving 10 mg empagliflozin and those receiving 25 mg empagliflozin, as shown in Fig. 5.

### Comparison of the ADEs Associated with 10 Versus 25 mg Empagliflozin During a Longer Follow-Up Period of ≥ 52 Weeks in Patients with T2DM

**Fig. 6:**
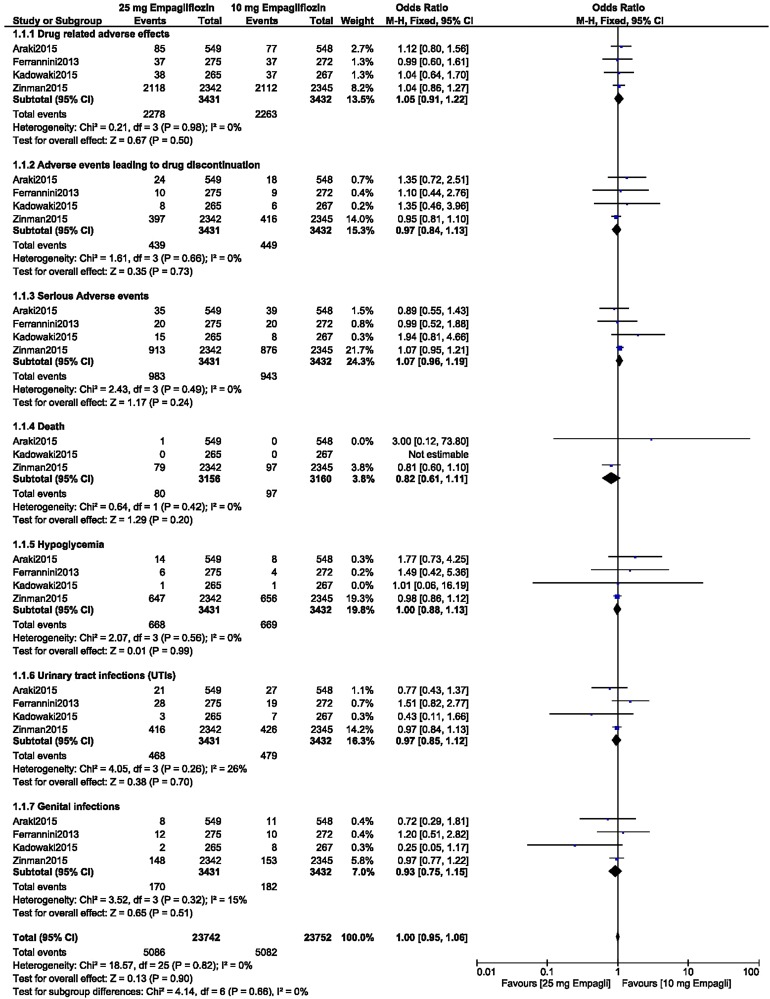
Adverse drug events associated with the treatment of patients with T2DM with 10 versus 25 mg empagliflozin during a relatively longer follow-up period of ≥ 52 weeks

Analysis of the data on treatment with 10 versus 25 mg empagliflozin during a relatively longer follow-up period of ≥ 52 weeks revealed that there were no significant differences in drug-related adverse effects (OR 1.05, 95% CI 0.91–1.22; *P* = 0.50, *I*^2^ = 0%), adverse events leading to drug discontinuation (OR 0.97, 95% CI 0.84–1.13; *P* = 0.73, *I*^2^ = 0%), serious adverse events (OR 1.07, 95% CI 0.96–1.19; *P* = 0.24, *I*^2^ = 0%), death (OR 0.82, 95% CI 0.61–1.11; *P* = 0.20, *I*^2^ = 0%), hypoglycemic episode (OR 1.00, 95% CI 0.88–1.13; *P* = 0.99, *I*^2^ = 0%), UTIs (OR 0.97, 95% CI 0.85–1.12; *P* = 0.70, *I*^2^ = 26%), and genital infections (OR 0.93, 95% CI 0.75–1.15; *P* = 0.51, *I*^2^ = 15%) between the T2DM patients receiving 10 mg empagliflozin and those receiving 25 mg empagliflozin, as shown in Fig. 6.

### Comparison of UTIs in Male Versus Female Patients with T2DM Treated with 10 and 25 mg Empagliflozin, Respectively

**Fig. 7:**
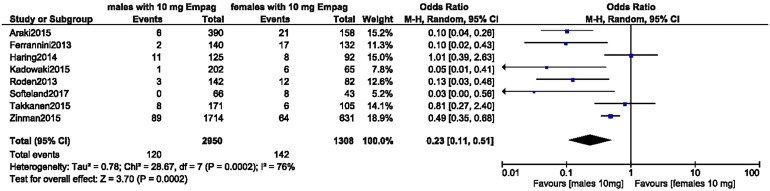
Urinary tract infections in male versus female patients with T2DM treated with 10 mg empagliflozin

**Fig. 8:**
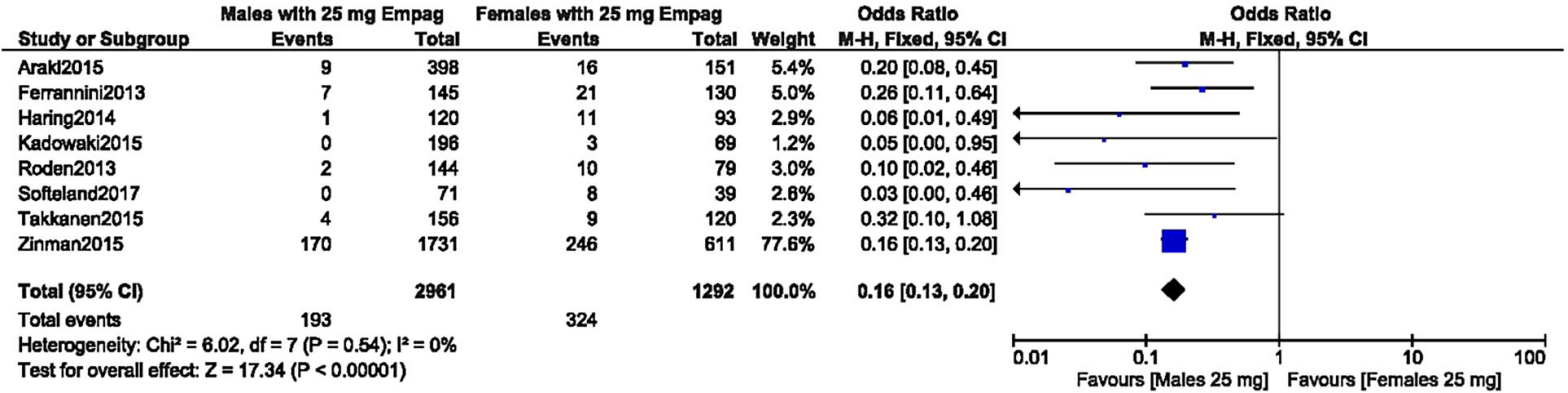
Urinary tract infections in male versus female patients with T2DM treated with 25 mg Empagliflozin

A separate analysis was carried out for UTIs in male versus female patients with T2DM treated with 10 mg empagliflozin. The analysis showed UTIs to be significantly higher in female patients (OR 0.23, 95% CI 0.11–0.51; *P* = 0.0002), as shown in Fig. 7. A second study was carried out for UTIs in male versus female patients with T2DM treated with 25 mg empagliflozin. The results again showed UTIs to be significantly higher in female patients (OR 0.16, 95% CI 0.13–0.20; *P* = 0.00001, *I*^2^ = 0%), as shown in Fig. 8.

#### Comparison of Genital Infections in Male Versus Female Patients with T2DM Treated with 10 and 25 mg Empagliflozin, Respectively

A separate analysis was carried out to compare genital infections in male versus female patients with T2DM treated with 10 and 25 mg empagliflozin, respectively. A comparison of these two treatment groups revealed that genital infections were significantly higher in female patients (OR 0.54, 95% CI 0.42–0.69; *P* = 0.00001) than in male patients (OR 0.40, 95% CI 0.18–0.88; *P* = 0.02).

### Sensitivity Analysis and Publication Bias

Sensitivity analyses produced consistent results. There was no significant difference in the results obtained when each trial was excluded one by one, and a new analysis was carried out each time.

Based on a visual inspection of the funnel plots which were generated, there was no evidence of publication bias, as observed across all the trials that assessed the adverse drug reactions in patients with T2DM receiving empagliflozin 10 mg versus those receiving empagliflozin 25 mg, as shown in Fig. 8a–c.

**Fig. 9:**
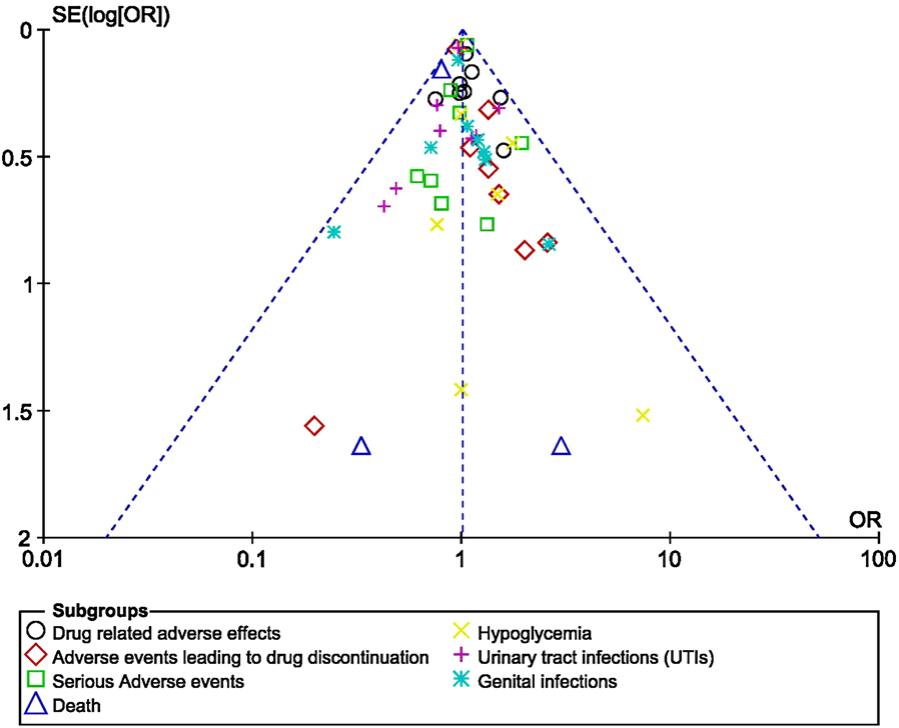

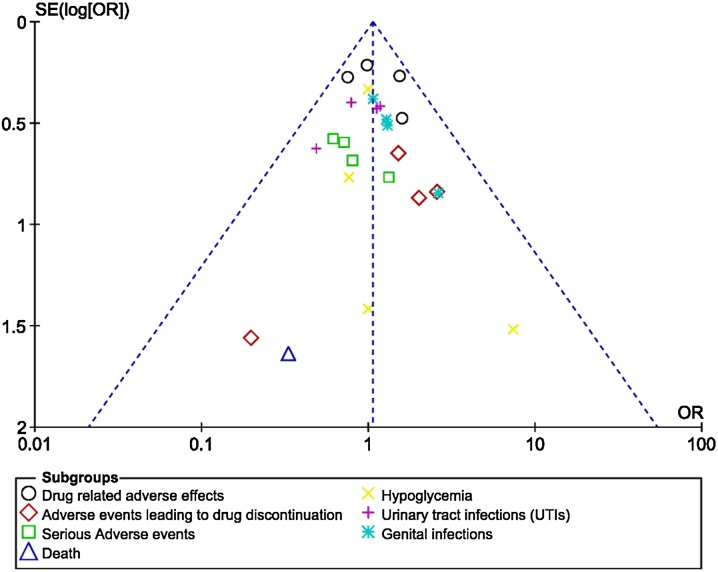

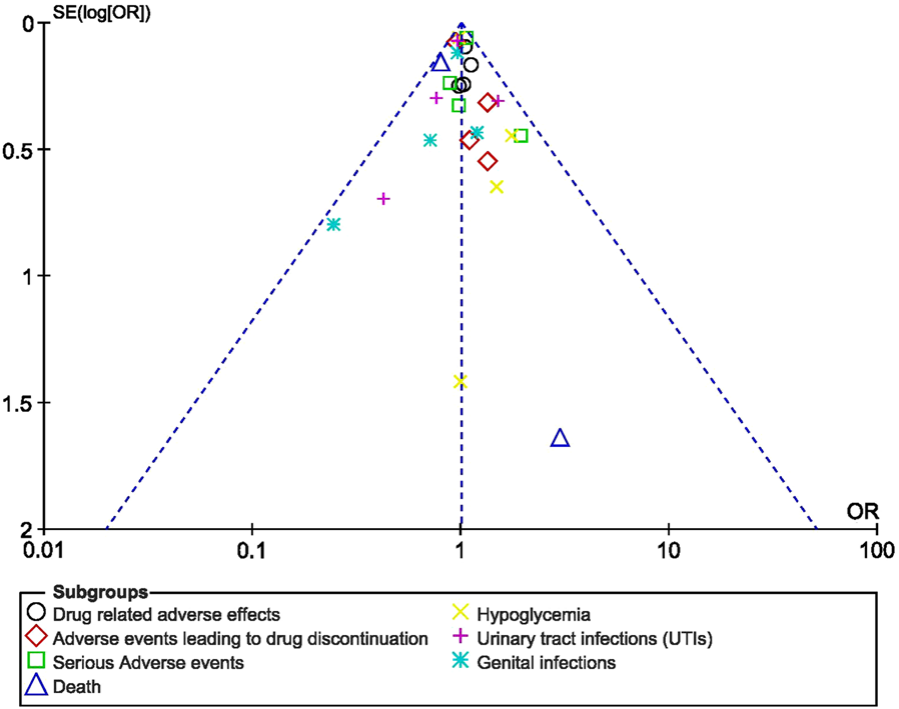
Funnel plots showing publication bias (**a**–**c**)

## Discussion

In this new era, it is high time to carry out research based not only on newer OHAs but also on the dosage of drugs to be prescribed.

Empagliflozin has been found to show beneficial effects in T2DM patients with cardiovascular disease. The EMPA-REG OUTCOME study reported an improvement in adverse cardiovascular outcomes in patients who were treated with empagliflozin [14]. T2DM patients at higher risk for cardiac problems and who were treated with empagliflozin had reduced composite cardiovascular outcomes and decreased mortality when this drug was added to the standard care [14].

Our analysis revealed that adverse drug outcomes were not significantly different between patients treated for T2DM with 10 or 25 mg empagliflozin. Similar results were obtained for both a shorter and a longer follow-up. However, when UTIs and genital infections were compared between male and female T2DM patients who received either 10 or 25 mg empagliflozin, both of these adverse events were significantly higher in the female patients.

The results of a recent analysis of pooled data from three clinical trials consisting of Japanese patients with T2DM also support the results of this current analysis in showing similar ADEs in patients who were treated with either dosage of empagliflozin [15]. Another double-blind extension of a phase III randomized controlled trial also showed similar results to our analysis whereby no significant difference was observed even after 76 weeks of treatment with empagliflozin 10 and 25 mg [16]. The results of other studies further support the findings of our analysis [17, 18].

Even though this is a systematic analysis of data which were obtained from recently published randomized controlled trials, the results may be novel compared to those of randomized trials due to the fact that male and female patients were also compared for UTIs and genital infections; such data were missing elements of the individual randomized controlled trials. Published research only shows data on UTIs and genital infections in males and females, but the data are never compared. However, in our analysis we were able to make a gender-based comparison.

### Novelty

This research study is novel in the following ways:

1. It is the first study to systematically compare the ADEs associated with treatment with 10 versus 25 mg empagliflozin in patients who were being treated for T2DM.
2. Several comparisons of empagliflozin treatment were made: (1) with empagliflozin as monotherapy; (2) with empagliflozin as add-on to other anti-diabetic medications; (3) with empagliflozin as add-on specifically to metformin, (4) during a short follow-up period, and (5) during a longer follow-up period.
3. Urinary tract infections and genital infections were also systematically compared in male versus female patients treated with 10 and 25 mg empagliflozin respectively.
4. The drug is a new, emerging OHA and it appears to have the potential to play a significant role in clinical medicine.

### Limitations

Few limitations were as followed:

1. Due to the limited number of patients especially with Empagliflozin mono-therapy, the results might further be confirmed in future trials with a larger population size.
2. The follow-up periods were not similar in each trial. This might also have had an effect on the results.
3. Other co-morbidities could have influenced the outcomes. However, co-morbidities at baseline were not often reported.

## Conclusions

Adverse drug events were not significantly different in patients with T2DM receiving 10 versus 25 mg empagliflozin as mono-therapy or as add-on to metformin or other anti-diabetic drugs during a shorter or longer follow-up period. However, genital and UTIs were more common in female patients with T2DM irrespective of empagliflozin dosage.

## References

[1] Bundhun PK, Janoo G, Huang F (2017). Adverse drug events observed in patients with type 2 diabetes mellitus treated with 100 mg versus 300 mg canagliflozin: a systematic review and meta-analysis of published randomized controlled trials. BMC Pharmacol Toxicol..

[2] Gerstein H, Jaeschke R (2016). Empagliflozin, cardiovascular outcomes, and mortality in type 2 diabetes mellitus. Dr. Hertzel Gerstein in an interview with Dr. Roman Jaeschke. Pol Arch Med Wewn.

[3] Natali A, Nesti L, Fabiani I, Calogero E, Di Bello V (2017). Impact of empagliflozin on subclinical left ventricular dysfunctions and on the mechanisms involved in myocardial disease progression in type 2 diabetes: rationale and design of the EMPA-HEART trial. Cardiovasc Diabetol..

[4] Tanaka A, Shimabukuro M, Okada Y, Taguchi I, Yamaoka-Tojo M, Tomiyama H, Teragawa H, Sugiyama S, Yoshida H, Sato Y, Kawaguchi A, Ikehara Y, Machii N, Maruhashi T, Shima KR, Takamura T, Matsuzawa Y, Kimura K, Sakuma M, Oyama JI, Inoue T, Higashi Y, Ueda S, Node K, EMBLEM Trial Investigators (2017). Rationale and design of a multicenter placebo-controlled double-blind randomized trial to evaluate the effect of empagliflozin on endothelial function: the EMBLEM trial. Cardiovasc Diabetol..

[5] Liberati A, Altman DG, Tetzlaff J (2009). The PRISMA statement for reporting systematic reviews and meta-analyses of studies that evaluate health care interventions: explanation and elaboration. BMJ.

[6] Higgins JP, Thompson SG, Deeks JJ (2003). Measuring inconsistency in meta-analyses. BMJ.

[7] Araki E, Tanizawa Y, Tanaka Y, Taniguchi A, Koiwai K, Kim G, Salsali A, Woerle HJ, Broedl UC (2015). Long-term treatment with empagliflozin as add-on to oral antidiabetes therapy in Japanese patients with type 2 diabetes mellitus. Diabetes Obes Metab.

[8] Ferrannini E, Berk A, Hantel S, Pinnetti S, Hach T, Woerle HJ, Broedl UC (2013). Long-term safety and efficacy of empagliflozin, sitagliptin, and metformin: an active-controlled, parallel-group, randomized, 78-week open-label extension study in patients with type 2 diabetes. Diabetes Care.

[9] Häring HU, Merker L, Seewaldt-Becker E, Weimer M, Meinicke T, Broedl UC, Woerle HJ, EMPA-REG MET Trial Investigators (2014). Empagliflozin as add-on to metformin in patients with type 2 diabetes: a 24-week, randomized, double-blind, placebo-controlled trial. Diabetes Care.

[10] Kadowaki T, Haneda M, Inagaki N, Terauchi Y, Taniguchi A, Koiwai K, Rattunde H, Woerle HJ, Broedl UC (2015). Efficacy and safety of empagliflozin monotherapy for 52 weeks in Japanese patients with type 2 diabetes: a randomized, double-blind, parallel-group study. Adv Ther..

[11] Roden M, Weng J, Eilbracht J, Delafont B, Kim G, Woerle HJ, Broedl UC, EMPA-REG MONO Trial Investigators (2013). Empagliflozin monotherapy with sitagliptin as an active comparator in patients with type 2 diabetes: a randomised, double-blind, placebo-controlled, phase 3 trial. Lancet Diabetes Endocrinol..

[12] Søfteland E, Meier JJ, Vangen B, Toorawa R, Maldonado-Lutomirsky M, Broedl UC (2017). Empagliflozin as add-on therapy in patients with type 2 diabetes inadequately controlled with linagliptin and metformin: a 24-week randomized, double-blind, parallel-group trial. Diabetes Care..

[13] Tikkanen I, Narko K, Zeller C, Green A, Salsali A, Broedl UC, Woerle HJ, EMPA-REG BP Investigators (2015). Empagliflozin reduces blood pressure in patients with type 2 diabetes and hypertension. Diabetes Care.

[14] Zinman B, Wanner C, Lachin JM, Fitchett D, Bluhmki E, Hantel S, Mattheus M, Devins T, Johansen OE, Woerle HJ, Broedl UC, Inzucchi SE, EMPA-REG OUTCOME Investigators (2015). Empagliflozin, cardiovascular outcomes, and mortality in type 2 diabetes. N Engl J Med.

[15] Shiba T, Ishii S, Okamura T, Mitsuyoshi R, Pfarr E, Koiwai K (2017). Efficacy and safety of empagliflozin in Japanese patients with type 2 diabetes mellitus: a sub-analysis by Body Mass Index and age of pooled data from three clinical trials. Diabetes Res Clin Pract.

[16] Roden M, Merker L, Christiansen AV, Roux F, Salsali A, Kim G, Stella P, Woerle HJ, Broedl UC, EMPA-REG EXTEND™ MONO Investigators (2015). Safety, tolerability and effects on cardiometabolic risk factors of empagliflozin monotherapy in drug-naïve patients with type 2 diabetes: a double-blind extension of a phase III randomized controlled trial. Cardiovasc Diabetol..

[17] Kaku K, Lee J, Mattheus M, Kaspers S, George J, Woerle HJ, EMPA-REG OUTCOME^®^ Investigators. Empagliflozin and cardiovascular outcomes in Asian patients with type 2 diabetes and established cardiovascular disease—results from EMPA-REG OUTCOME^®^. Circ J 2017;81(2):227–234. 10.1253/circj.CJ-16-1148 28025462

[18] Gupta S, Shaikh S, Joshi P, Bhure S, Suvarna V (2017). Long-term efficacy and safety of empagliflozin monotherapy in drug-naïve patients with type 2 diabetes in indian subgroup: results from a 76-week extension trial of phase III, double-blind, randomized study. Indian J Endocrinol Metab..

